# Regional disparities in breast cancer healthcare in Japan: REAL-BC study

**DOI:** 10.1007/s12282-025-01739-x

**Published:** 2025-08-02

**Authors:** Hiroshi Kitagawa, Kazuki Fukuzawa, Masaki Tanaka

**Affiliations:** Medical Department, AstraZeneca K.K, Grand Front Osaka, Tower B, 3-1, Ofuka-Cho, Kita-Ku, Osaka 530-0011 Japan

**Keywords:** Breast cancer, Japan, Real-world, Healthcare, Regional variability

## Abstract

**Background:**

Since 2007, the Basic Plans for Promoting Cancer Control (BPPCCs) have sought to enhance cancer care at designated cancer care hospitals (DCCHs) and implement population (PBCR)- and hospital (HBCR)-based cancer registries, among other activities. However, the impact of the BPPCCs on breast cancer care in Japan remains uncertain. This study sought to reveal the reality of regional disparities in the proportion of breast cancer patients' initial visits to DCCHs and the functionality of breast cancer in DCCHs.

**Methods:**

We obtained data from the PBCR and HBCR in Japan, as well as administrative healthcare claims data (JMDC claims data), and data published by clinical societies in Japan for the period 2018 to 2021. We conducted descriptive analyses to determine the proportions of patients who received initial treatment for breast cancer at a DCCH. We also examined the quality of care in terms of staffing and functions.

**Results:**

In 2020, out of 79,062 breast cancer patients registered in the HBCR, 57.7% started initial treatment at a DCCH (range across 47 prefectures: 15.5%–89.8%) in Japan. The proportion of patients who visited a DCCH for initial treatment increased from 53.8% (2018) to 57.7% (2020). The median proportion of DCCHs certified by the Japanese Breast Cancer Society (JBCS) among the 47 prefectures was 62.50% (range: 16.7%–100.0%). The median number of patients per JBCS-certified specialist was 77.40 (range: 37.6–142.0). The proportions of DCCHs with claims for breast cancer-related service fees were 15.2% (range: 0.0%–50.0%) for cancer genome profiling tests, 62.0% (range: 0.0%–100.0%) for cancer *BRCA1/2* genetic tests, and 92.8% (range: 60.0%–100.0%) for patient support system enhancement.

**Conclusion:**

We found regional disparities in the initial treatment and medical services for breast cancer care in Japan. The findings uncover opportunities to enhance the treatment of breast cancer in Japan. We anticipate that our data will be utilized as a valuable resource and as a key input for informing policy development tailored to the specific characteristics of the region and for designing programs to address the different needs of each prefecture.

**Supplementary Information:**

The online version contains supplementary material available at 10.1007/s12282-025-01739-x.

## Introduction

### Cancer burden and cancer control act in Japan

Cancer has been the leading cause of death in Japan since 1981, when it accounted for 142.0 per 100,000 deaths, reaching 316.1 per 100,000 deaths in 2022 [1], an increase that could be attributed to the aging of the Japanese population, placing a massive burden on the healthcare system.

To address cancer care in Japan, the Cancer Control Act led to the First Basic Plan for Promoting Cancer Control (BPPCC) in 2007 [2]. An important component was implementation of a cancer registry. More recently, the Third BPCC [2, 3] introduced designated cancer care hospitals (DCCHs) for care delivery, along with hospital-based cancer registries (HBCRs) and nationwide population-based cancer registries (PBCRs). By 2021, the HBCR covered approximately 71.7% of patients treated for cancer in hospitals, and 52.5% of patients with cancer were treated at DCCHs [4].

### Current state of breast cancer in Japan

Breast cancer is the most common type of cancer in women; Japanese projections estimated a prevalence of 94,300 women and 15,600 deaths due to breast cancer in 2022 [5]. The treatment modality chosen for an individual patient is usually made according to the patient’s stage and clinical characteristics at diagnosis [6, 7] by a multidisciplinary team of healthcare professionals. However, the composition of the multidisciplinary team, the hospital approach, and the role of DCCHs may differ among regions. These factors may lead to differences or disparities in breast cancer care [2, 3]. Although the overall proportion of patients registered at DCCHs has been reported [4], there is little clarity regarding the proportions of visits in each prefecture. Therefore, it is essential to investigate and understand the extent of potential regional disparities to ensure equitable access and optimize the delivery of care.

The Japanese Breast Cancer Society (JBCS) implemented the National Breast Cancer Registry as a subset of the National Clinical Database to monitor the trends in the characteristics and treatment of breast cancer since 2012, including quality of care and compliance with guidelines, and some studies have utilized this registry to examine trends in treatment patterns and survival [8, 9]. To date, however, such registries have not been well integrated with or used disease-specific information, such as the operational aspects of the system and how they affect outcomes, in the context of the BPPCCs. Therefore, studies are needed to address the limitations of prior work to better understand the management of breast cancer, and identify potential disparities that could be targeted in future activities. This is relevant in the context of the Japanese health structure. This includes a free access system under the universal health insurance system, without restrictions on where people receive their care, allowing people to visit any medical institution in Japan.

In this study, we primarily aimed to describe the prefectural differences in the ratio of breast cancer patients visiting DCCHs for their initial treatment in 2020 (see ESM Table 1 in Online Resource 1 for a detailed list of objectives). Our secondary objective was to describe the prefectural reality regarding the functions, staffing, and medical services of DCCHs for breast cancer, as described in ESM Table 2 (Online Resource 1). As exploratory objectives, we investigated the regional differences in the ratio of breast cancer patients visiting DCCHs for their initial treatment in 2020 and the trends of prefectural differences in the ratio of breast cancer patients visiting DCCHs for their initial treatment per calendar year from 2018 to 2020 in secondary healthcare service area units.

## Methods

### Data sources

**Fig. 1:**
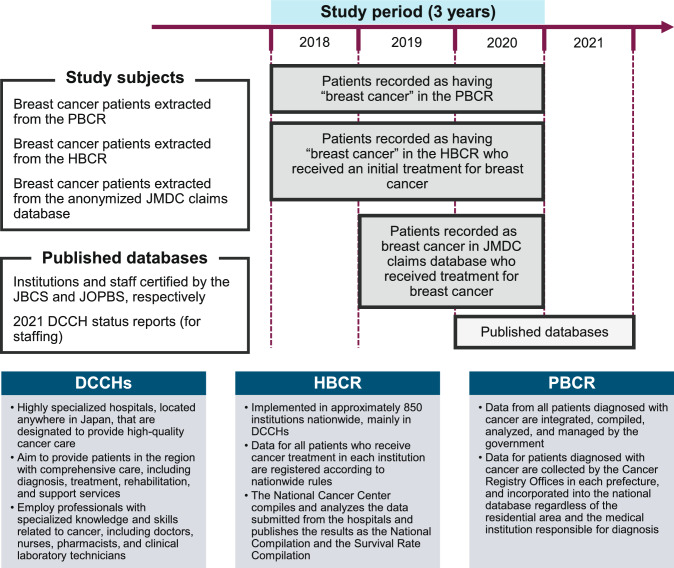
Overview of data sources and definitions of the DCCH, HBCR, and PBCR. *DCCH* designated cancer care hospital, *HBCR* hospital-based cancer registry, *JBCS* Japanese Breast Cancer Society, *JOPBS* Japan Oncoplastic Breast Surgery Society, *PBCR* population-based cancer registry

We used the following data sources for Japan: the PBCR and HBCR (collated by the National Cancer Center of Japan), the JMDC claims database (JMDC Inc., Tokyo, Japan), and public data. Fig. 1 depicts the study period and data sources. We obtained public data from the sources listed in ESM Table 3 (Online Resource 1). HBCR data were provided based on legal regulations and were prepared and modified by the authors for publication.

### Patients

We retrieved patients from the databases for the specified study periods. For the PBCR, we retrieved data for patients diagnosed with breast cancer in each calendar year from 2018 to 2020. For the HBCR, we used data for patients initially treated with breast cancer and registered with the HBCR in each calendar year from 2018 to 2020. The HBCR included data from DCCHs and non-DCCHs. Therefore, we extracted the subset of patients who received initial treatment at DCCHs from the HBCR.

For the PBCR and HBCR, we used data through 2020 because these were the latest available data at the time of planning the study, and this year they represent the mid-year of the Third BPPCC (2017–2022).

For the JMDC claims database, we retrieved patients with a diagnosis of breast cancer (International Classification of Diseases, 10th revision, codes C50 or D05; listed in ESM Table 4 [Online Resource 1]) who received any breast cancer-related treatment in the calendar years 2020 and 2021 (summarized in ESM Table 5 [Online Resource 1]). We used a previously described algorithm to identify patients with breast cancer [10, 11].

We defined initial cancer treatment in the HBCR as any treatment performed after the initial diagnosis of the tumor, out of all cancer treatments intended to reduce or excise the tumor, for operational necessity. The scope of the treatment performed after the initial diagnosis was the treatment described in the treatment plan. If follow-up was planned or the patient died before treatment, the follow-up observation was considered as the initial treatment.

### Data analyses

All data were analyzed using descriptive statistics by presenting the number, frequency, and percent of patients, or the mean, standard deviation, median, and range, depending on the type of data. The formulas used to calculate the specific study outcomes are described in ESM Table 1 (Online Resource 1). We did not impute missing values, perform statistical comparisons, or calculate p-values. SAS Viya version 3.5 (SAS Institute Inc., Cary, NC, USA) was used for the analyses.

## Results

### Available data

A total of 430, 444, and 448 DCCHs that cared for at least one breast cancer patient were identified in 2018, 2019, and 2020, respectively (ESM Table 6 [Online Resource 1]). The JMDC claims database included data for 389 DCCHs (ESM Table 7 [Online Resource 1]).

### Initial treatment in 2020

**Fig. 2:**
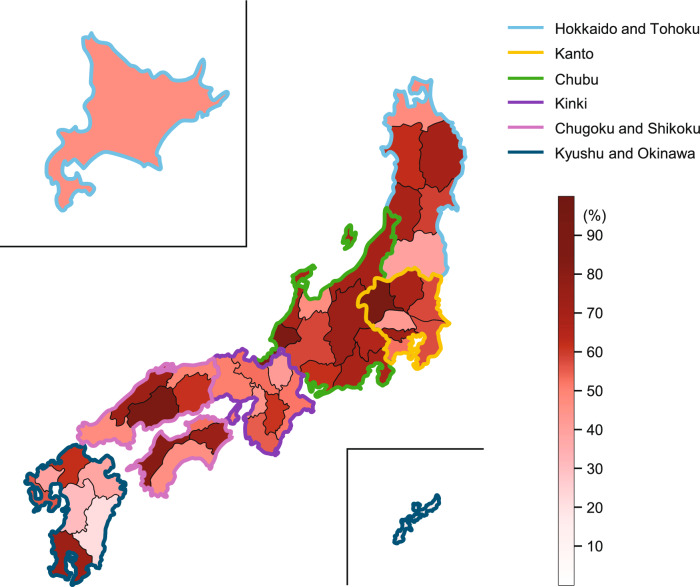
Heatmap of the ratios of breast cancer patients who visited DCCHs for initial treatment by prefecture in 2020. The values in each prefecture are given in ESM Table 8 (Online Resource 1). *DCCH* designated cancer care hospital

In 2020, a total of 103,744 breast cancer patients were registered in the PBCR and 79,062 (76.2%) were registered in the HBCR and received initial treatment. Of these, 59,891 (57.7%) started initial treatment at a DCCH. Data for individual prefectures are shown in the heatmap in Fig. 2 and ESM Table 8 (Online Resource 1). The proportion ranged from 15.5% to 89.8% and was ≥ 80% in 4 prefectures and < 40% in 6 prefectures.

### Differences among prefectures in the function, staffing, and status of medical services at DCCHs for breast cancer patients in 2020

**Fig. 3:**
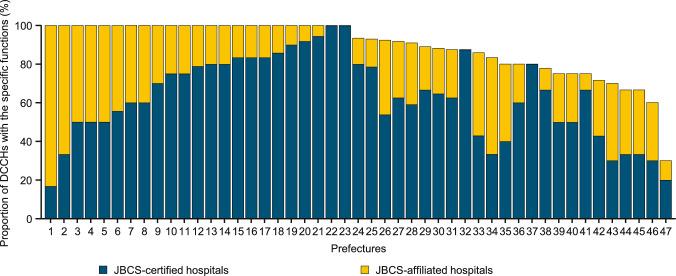
Bar chart showing the proportion of DCCHs designated as certified or affiliated by the JBCS. Prefectures are numbered the same in Figs. 3 and 4b. *DCCH* designated cancer care hospital, *JBCS* Japanese Breast Cancer Society

We assessed the function of identified DCCHs (*n* = 448) in terms of certification or affiliation by relevant societies, and the data are shown in Table 1. In 2020, the median (range by prefecture) proportion of DCCHs certified by the JBCS was 62.50% (16.7%–100.0%) and 25.0% (0.0%–83.3%) were affiliated with the JBCS. The distribution of breast cancer-related functions, in terms of the proportions of JBCS-certified hospitals and JBCS-affiliated hospitals, also varied among prefectures (Fig. 3). All of the prefectures had at least one JBCS-certified hospital, highlighting the availability of breast cancer care in each prefecture. The proportion of hospitals certified by the Japan Oncoplastic Breast Surgery Society (JOPBS) was 71.40% (16.7%–100.0%). Per Cancer Genome Medicine designations, the median (range by prefecture) proportions of DCCHs designated as core, hub, and affiliated hospitals were 0% (0.0%–12.5%), 0% (0.0%–20.0%), and 33.3% (0.0%–80.0%), respectively (Table 1). Although there was at least one Cancer Genome Medicine-designated hospital in each prefecture, 21 prefectures lacked both core and hub hospitals.

**Table 1:** Summary of the function, staffing, and medical services utilized at DCCHs across the 47 prefectures in Japan

	*N*	Mean	Minimum	Median	Maximum
Function (proportion of DCCHs)					
JBCS-certified hospitals	47	62.57%	16.7%	62.50%	100.0%
JBCS-affiliated hospitals	47	26.58%	0.0%	25.00%	83.3%
JOPBS implantation-certified hospitals	47	65.65%	16.7%	71.40%	100.0%
Cancer Genome Medicine core hospitals	47	1.61%	0.0%	0.00%	12.5%
Cancer Genome Medicine hub hospitals	47	6.25%	0.0%	0.00%	20.0%
Cancer Genome Medicine-affiliated hospitals	47	33.86%	0.0%	33.30%	80.0%
Staffing (number of breast cancer patients per specialist)					
JBCS-certified physicians	47	80.90	37.6	77.40	142.0
JOPBS-certified responsible physicians and performing physicians	47	53.34	22.2	51.20	87.9
JSMO-certified medical oncologists	47	75.53	27.3	68.10	174.8
JSP-certified pathologists	47	56.10	23.8	55.90	102.5
JASTRO/JRS-certified radiologists	46	69.32	34.1	70.75	145.5
JSPHCS-certified cancer specialist pharmacists	44	133.52	35.2	110.30	366.9
JNA-certified breast cancer nurses	45	236.43	71.0	237.50	398.6
Medical services (proportion of DCCH)					
Cancer patient guidance and management fee (I)	47	60.75%	0.0%	60.00%	100.0%
Cancer patient guidance and management fee (Ro)	47	51.35%	0.0%	50.00%	100.0%
Cancer patient guidance and management fee (Ha)	47	65.11%	0.0%	71.40%	100.0%
Cancer patient guidance and management fee (Ni)	47	23.06%	0.0%	22.20%	66.7%
Cancer genome profiling test fee	47	13.15%	0.0%	11.10%	50.0%
Cancer *BRCA1/2* genetic test fee	47	58.76%	0.0%	60.00%	100.0%
Genetic counseling fee	47	33.21%	0.0%	33.30%	72.4%
Patient support system enhancement fee	47	90.99%	60.0%	100.00%	100.0%
Cancer patient rehabilitation fee	47	53.28%	0.0%	58.30%	100.0%
Lymphedema combination therapy fee	47	15.43%	0.0%	12.50%	66.7%
Cancer treatment coordination planning fee 1	47	27.22%	0.0%	20.00%	87.5%

*DCCH* designated cancer care hospital, *JBCS* Japanese Breast Cancer Society, *JNA* Japanese Nursing Association, *JOPBS* Japan Oncoplastic Breast Surgery Society, *JSMO* Japanese Society of Medical Oncology, *JSP* Japanese Society of Pathology, *JSPHCS* Japanese Society of Pharmaceutical Health Care and Sciences, *JASTRO/JRS* Japanese Society for Radiation Oncology/Japan Radiological Society

**Fig. 4:**
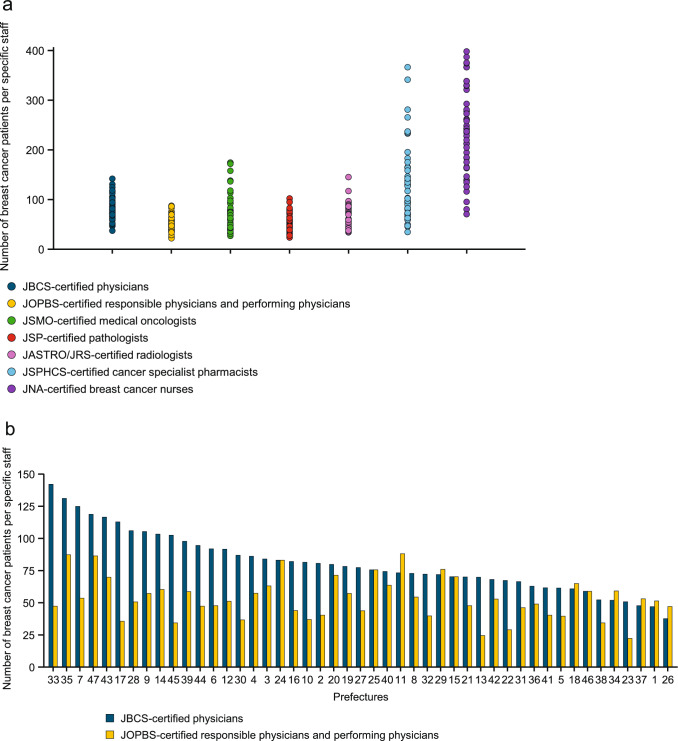
Staffing at DCCHs. **a** Dot plot of the number of breast cancer patients per specialist. **b** Bar chart of the number of patients per JBCS-certified physician and number of patients per JOPBS-certified physician in each prefecture. Prefectures are numbered the same in Figs. 3 and 4b. *DCCH* designated cancer care hospital, *JASTRO/JRS* Japanese Society for Radiation Oncology/Japan Radiological Society, *JBCS* Japanese Breast Cancer Society, *JNA* Japanese Nursing Association, *JOPBS* Japan Oncoplastic Breast Surgery Society, *JSMO* Japanese Society of Medical Oncology, *JSP* Japanese Society of Pathology, *JSPHCS* Japanese Society of Pharmaceutical Health Care and Sciences

Regarding staffing, the number of specialists and the number of breast cancer patients per specialist type are displayed in Fig. 4a. The median number of breast cancer patients per specialist (Table 1) was 77.40 (range by prefecture: 37.6–142.0) for JBCS-certified physicians, 51.20 (22.2–87.9) for JOPBS-certified physicians, 68.10 (27.3–174.8) for Japanese Society of Medical Oncology (JSMO)-certified medical oncologists, 55.90 (23.8–102.5) for Japanese Society of Pathology (JSP)-certified pathologists, 70.75 (34.1–145.5) for Japanese Society for Radiation Oncology/Japan Radiological Society (JASTRO/JRS)-certified radiologists, 110.30 (35.2–366.9) for Japanese Society of Pharmaceutical Health Care and Sciences (JSPHCS)-certified cancer specialist pharmacists, and 237.50 (71.0–398.6) for Japan Nursing Association (JNA)-certified breast cancer nurses. There were no JASTRO/JRS-certified radiologists in one prefecture, JSPHCS-certified cancer specialist pharmacists in three prefectures, and JNA-certified breast cancer nurses in two prefectures. The number of patients per JBCS-certified physician and the number of patients per JOPBS-certified responsible physician or performing physician in individual prefectures are presented in Fig. 4b.

**Fig. 5:**
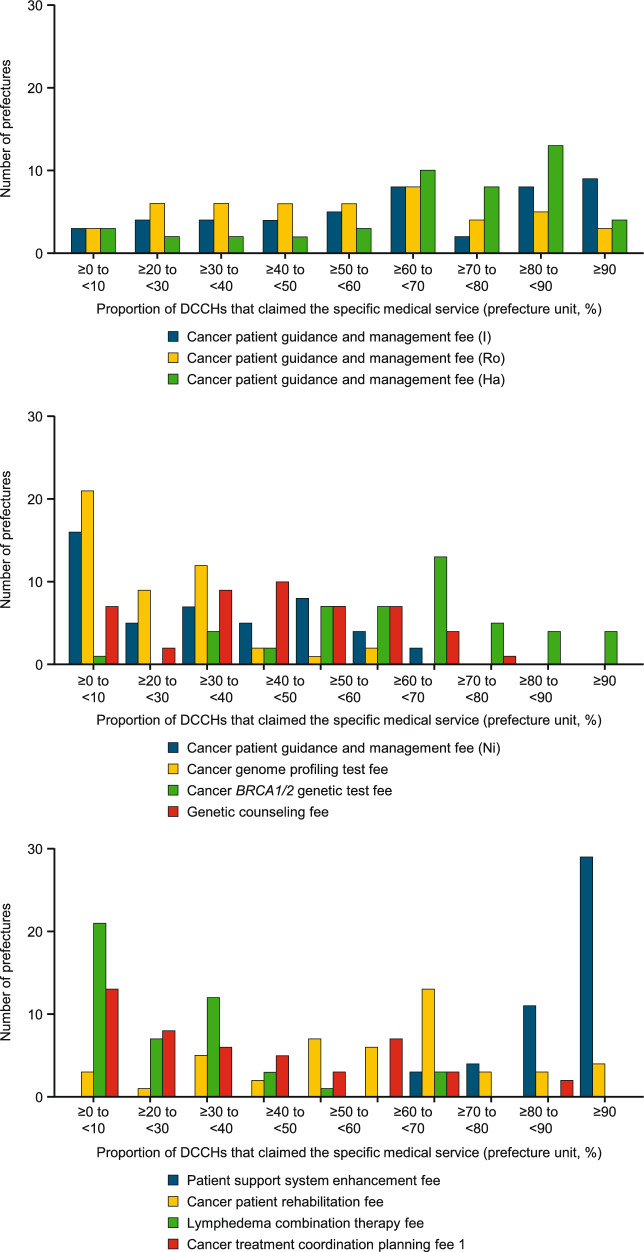
Histograms of proportions of DCCHs claiming specific medical services. *DCCH* designated cancer care hospital

Medical services were assessed using relevant fees (see ESM Table 2 [Online Resource 1] for definitions) and the results are presented in Table 1 and Fig. 5 for 389 DCCHs in 2020. Cancer patient guidance and management fees were recorded at 60.00% (median) of DCCHs (range by prefecture: 0.0%–100.0%) for fee “I” (discussion of the treatment strategy), 50.00% (0.0%–100.0%) for fee “Ro” (interviews to reduce psychological anxiety), 71.40% (0.0%–100.0%) for fee “Ha” (discussion of need for anticancer agents), and 22.20% (0.0%–66.7%) for fee “Ni” (discussion of the need for genetic testing). Cancer genome profiling tests, cancer *BRCA1/2* genetic tests, and genetic counseling were accessed at 11.10% (range by prefecture: 0.0%–50.0%), 60.00% (0.0%–100.0%), and 33.30% (0.0%–72.4%) of DCCHs, respectively. Other services included patient support system enhancement, cancer patient rehabilitation, lymphedema combination therapy, and cancer treatment coordination planning, which were accessed at 100.00% (range by prefecture: 60.0%–100.0%), 58.30% (0.0%–100.0%), 12.50% (0.0%–66.7%), and 20.00% (0.0%–87.5%) of DCCHs, respectively.

### Time trends in the proportions of breast cancer patients visiting DCCHs for initial treatment

**Fig. 6:**
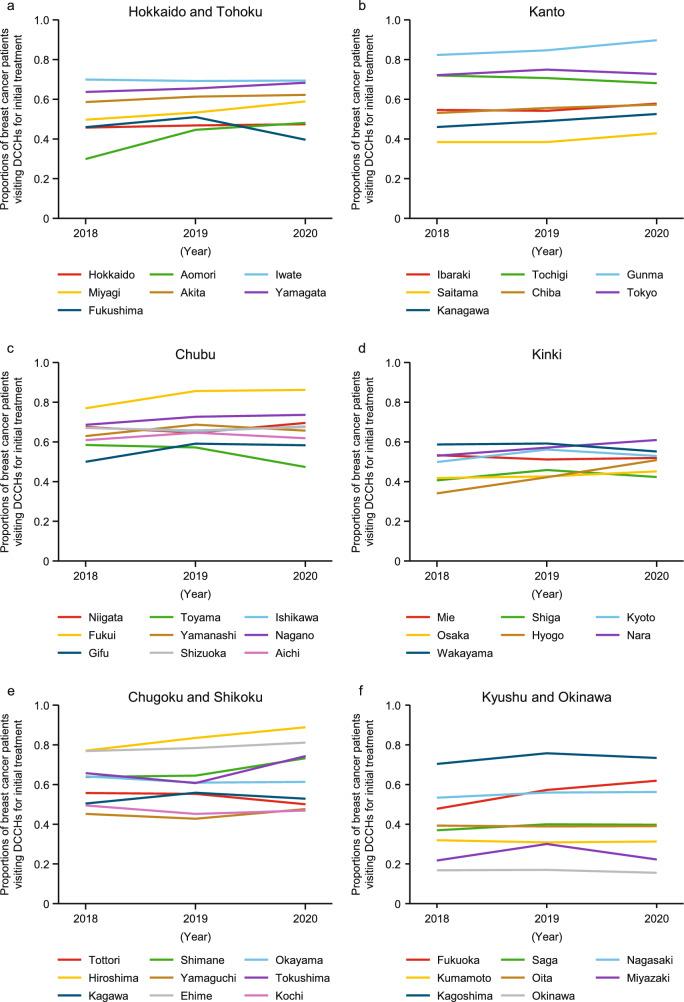
Proportions of breast cancer patients visiting DCCHs for initial treatment, by prefecture per calendar year. **a** Hokkaido and Tohoku. **b** Kanto. **c** Chubu. **d** Kinki. **e** Chugoku and Shikoku. **f** Kyushu and Okinawa. *DCCH* designated cancer care hospital. *DCCH* designated cancer care hospital

Fig. 6 shows the time trends in proportions of breast cancer patients visiting DCCHs for initial treatment, by prefecture per calendar year. ESM Table 9 (Online Resource 1) presents the descriptive analysis of data across the 47 prefectures. Overall, the proportion of patients who visited DCCHs for initial treatment increased from 53.8% in 2018 to 57.7% in 2020.

## Discussion

To our knowledge, this was the first cross-sectional study to report on regional disparities in breast cancer care by focusing on DCCHs in Japan. Our analysis revealed some variability in the outcomes among prefectures in Japan with our estimates suggesting that just over half (57.7%) of breast cancer patients received initial treatment at a DCCH in 2020, a value that varied considerably among the prefectures, ranging from 15.5% to 89.8%, as illustrated in Fig. 2.

The variability among prefectures may be due to two factors: the difference in the proportion of patients registered in the HBCR among all breast cancer patients registered in the PBCR, and the difference in the proportion of patients who visited a DCCH for their initial treatment (ESM Fig. 1 [Online Resource 1]). The median proportion of patients registered in the HBCR out of all breast cancer patients was < 50% in three prefectures where most patients visited facilities not participating in the HBCR. The median percentage of patients registered in the HBCR who visited a DCCH was < 60% in five prefectures. In Okinawa, one of these prefectures, although the percentage of patients registered in the HBCR among all breast cancer patients was 63.4%, the percentage of patients registered in the HBCR who visited a DCCH was just 24.4%. According to the annual HBCR report, this is due to the geography of Okinawa, which consists of multiple islands, and the fact that breast cancer is mainly treated at “breast clinics,” which cannot participate in the HBCR [12]. Given this context, any measures aimed at improving the quality of medical care in the future should consider the actual condition and clinical situation in individual regions. This is particularly important because Japan is a country with a rapidly aging population, especially in rural areas. Therefore, it is crucial to ensure that people have access to medical care, particularly in underpopulated or remote areas, through measures such as shuttle services, telemedicine, or funded accommodation. For this, we may look to other countries like Australia, which also exhibits marked variation and disparities in breast cancer care between regional and rural areas. In particular, rural patients frequently encountered delayed treatment and had lower rates of breast-conserving surgery, which was at least partly related to limited access to specialists, travel costs, and inadequate care coordination [13]. In an effort to address such disparities, Australia has implemented initiatives to ensure people living in rural or remote areas can access the same quality of care as people in metropolitan areas. These programs include facilitating finding cancer specialists and hospitals and offering subsidized accommodation and patient-assisted travel schemes [14, 15]. In summary, when interpreting the variability among prefectures, it is important to consider that breast cancer care is provided according to regional features, including geographical characteristics and demographic trends, and any prefectural measures should consider these factors.

We also looked at the trends in the proportions of breast cancer patients who visited DCCHs for their initial treatment over time and found a continuous increase between 2018 and 2020 overall (from 53.8% in 2018 to 56.6% in 2019 and 57.7% in 2020). This trend might be due to a variety of measures, including the implementation of the recommendations of the Third BPPCC, particularly enhanced capability and availability of DCCHs. While most prefectures demonstrated similar trends, a small decline over this period occurred in some prefectures. We believe that this was driven by the COVID-19 pandemic, which potentially interfered with breast cancer diagnosis and treatment in terms of resources or the capacity for delivering breast cancer care as part of COVID-19-related risk management in 2020. Supporting this possibility, a prior study using the HBCR revealed reductions in cancer cases, cancer screening, and cancer detection rates in 2020 compared with those in 2016–2019 [16]. Additionally, the rate of breast cancer diagnosis was reported to decline in 2020 compared with 2019, with reductions in services such as breast biopsy [17] and decreased cancer screening [18]. Another report showed that the COVID-19 pandemic was associated with a diagnosis of more aggressive and more advanced disease following the suspension of breast cancer screening services [19]. In view of these findings, it is possible that the proportion of patients who received initial treatment at a DCCH in 2020 could have been affected by the COVID-19 pandemic. Overall, we should interpret these trends cautiously.

Another objective of our study was to examine the function, staffing, and medical services utilized at DCCHs. We noted some variability among the prefectures. In terms of the functions of the DCCHs, although all 47 prefectures had at least one JBCS-certified institution and at least one JOPBS-certified institution, the numbers varied among prefectures. The proportions of hospitals classified as Cancer Genomic Medicine core, hub, and affiliated were 2.7%, 7.1%, and 34.8%, respectively. Notably, 21 prefectures lacked both core- and hub-designated hospitals, although all prefectures had at least one Cancer Genome Medicine-designated (core, hub, or affiliated) hospital. From a staffing perspective, the data from 2020 indicated there were 769 specialists certified by the JBCS, 1,159 certified by or affiliated with the JOPBS, and 231 breast cancer nurses certified by the JNA. The median numbers of specific staff in DCCHs per prefecture were 77.40, 51.20, and 237.50, respectively. These values varied greatly among the prefectures.

Certified institutions, specialists, and specialized nurses are given functions/qualifications according to the requirements established by each society. For breast cancer, two societies have established requirements related to hospital function, and three societies have established requirements related to personnel. Utilizing an independent breast cancer registry combined with the quality indicators set by the JBCS has contributed to improved patient outcomes [8, 20]. However, there appears to be limited cooperation among societies, DCCHs, and local governments in terms of certifying institutions and personnel with the aim of improving the quality of community medical care along with the BPPCCs. In Europe, the initiative “Transforming Breast Cancer Together” was implemented in 2017. In this initiative, the European Parliament and several academic societies involved in breast cancer care, including the European Society of Breast Cancer Specialists (EUSOMA), the European Society of Surgical Oncology, and the European Breast Cancer Coalition, are collaborating to improve breast cancer care [21]. Other activities driven by EUSOMA, as an example, include performance monitoring using quality indicators [22] and certification of breast units [23]. Differences in healthcare policies and financial resources may affect healthcare facilities and personnel support, and urban areas may attract more specialized staff due to better infrastructure. Therefore, further cooperation between cancer care policymakers and societies is desired to further support the functions of institutions and deploy specialized personnel related to breast cancer care. One effective approach can be seen in Scotland [24], which aims to make cancer services more efficient and effective by emphasizing patient feedback and user experience to maintain and improve the performance of standard cancer care across the country. The introduction of a similar approach in Japan could further increase the number of patients visiting DCCHs for breast cancer treatment.

Although the data only covered approximately 14% of the Japanese population and were coupled with other limitations, and the information gained should not be conclusive, we tried to identify potential issues that could be addressed from the health services perspective by analyzing the proportions of patients with relevant medical fees for services related to breast cancer in the study period. The percentages of patients who received cancer patient management and guidance fees, which correspond to the provision of care-related information or interviews by healthcare professionals, did not reach 100%. In fact, these proportions were 0% in three prefectures. The percentage of patients with a cancer patient management and guidance fee (Ni), which is a category related to genetic testing, was 22.20%. Meanwhile, the percentages of patients with the *BRCA1/2* genetic test fee, genetic counseling, and cancer genome profiling test fees were 60.00%, 33.00%, and 11.10%, respectively. Although services related to *BRCA* testing were frequently accessed, relatively few patients received services related to genetic counseling. This may be because the number of institutions for Cancer Genomic Medicine covers less than half of DCCHs. Regarding the variability of all the health services examined here, further research is needed to better understand these findings. It may be necessary to review the calculation requirements to ensure that they are consistent with the medical reality because of differences in staffing availability between hospitals in rural and urban locations.

Our study captured the progressive implementation of the Third BPPCC using public data and claims data from the perspective of breast cancer. In an interim report [25], it was noted that overall cancer care has improved following the introduction of DCCHs, but the progress varied among regions and medical institutions [25]. In fact, we have revealed some variabilities in the function and staffing assignments related to breast cancer care, not just its treatment, among prefectures. Ongoing activities to improve the quality of cancer care include those driven by the PDCA Cycle Forum [26]. Additionally, discussions between local government departments and DCCHs provide actions to implement based on data reported to the Base Hospital Liaison Council and core hospitals for cancer care coordination [27]. Additionally, the JBCS independently monitors the quality of breast cancer care using a patient registration system and quality indices, including postoperative radiotherapy and the proportion of patients undergoing HER2 testing. The availability of these services at DCCHs/base hospitals and the number of certified healthcare professionals represent how well each prefecture has incorporated the clinical guidelines developed by the JBCS and other relevant societies [8, 9]. However, some issues remain. For example, the currently available information is not fully utilized, the municipality participants only have observer status, and societies have limited involvement. Okuyama and Higashi shed light on the usefulness of and challenges associated with linking the HBCR and diagnostic procedure combination-based information [28]. They mentioned that it was useful to analyze such information with the aim of supplementing cancer registry information and gaining a more detailed understanding of the clinical reality, but they also highlighted the need to improve the process for utilizing the information, such as simplifying the approval system and introducing digital tools [29]. Therefore, it is hoped that sharing disease-specific information that better reflects the regional conditions with local healthcare professionals, government administrators, and other relevant stakeholders, such as society committees, will help us to identify and resolve problems, and ultimately achieve uniform accessibility to reduce disparities. In addition, providing patients with appropriate information could help to reduce disparities from the perspective of patients.

### Limitations

There are some important limitations of this study that may impact the generalizability of this study. Based on 2020 data, the HBCR comprised 79,062 patients, including 59,891 (57.7%) of patients who visited a DCCH for initial treatment of breast cancer. However, 103,744 patients with breast cancer were recorded in the PBCR. Therefore, the HBCR does not include all patients registered in the PBCR. The JMDC claims database is a large database compiling administrative data (healthcare claims) for over 17 million people in Japan, approximately 14% of the total Japanese population [30]. However, it has some limitations that influence the generalizability. For example, it is primarily composed of working-age people with a mean age of 35.7 years [31]. It cannot trace patients whose insurance category changes (e.g. due to a change of employer or stopping work) and some patients may be duplicated following the change in the insurance category. Furthermore, only a small proportion of patients are ≥ 65 years and none are aged ≥ 75 years old, indicating an underrepresentation of elderly patients. Other possible limitations of our study include the inability to track patients across multiple data sources, which could prevent us from understanding the reason(s) for the disparities in regions. If the HBCR could be linked to the JMDC claims database, it may be possible to collate more information on the stage of cancer in relation to treatment, and more accurately evaluate the facilities available at DCCHs. Finally, we sought to elucidate the real-world situation in Japan and identify possible gaps, not to propose specific solutions or strategies.

## Conclusions

In conclusion, this study has identified some regional disparities in the rate of breast cancer patients who visited DCCHs for initial treatment in Japan in 2020, as well as the function, staff assignments, and status of medical services at DCCHs. Of note, data were retrieved from several sources: the HBCR covers a large proportion of patients with breast cancer, and is considered comprehensive and reliable; the data obtained from academic societies reflect the actual function and staff assignment; and the claims database reflects the actual status of medical care based on the insurance claims, although it contains no data for elderly people. We anticipate that our data will be utilized as a valuable resource and one of the key inputs for informing policy development tailored to the specific characteristics of the region and for designing programs to address the different needs of each prefecture. Furthermore, we believe the insight obtained in this study is important for further integration of national cancer control activities and activities performed by the JBCS or other relevant key stakeholders. Additionally, further utilization of clinical data and collaboration with disease-specific societies, particularly regarding the training and allocation of specialists, will be useful for future improvement in breast cancer care. Considering the exploratory nature of this study, further analyses with appropriate statistical modeling are warranted to confirm and extend our findings, including future trends and prefecture-level differences. Overall, our results should be useful to develop hypotheses for such studies and to design appropriate strategies to improve the quality of breast cancer care in Japan in the future.

## Supplementary Information

Below is the link to the electronic supplementary material.

Supplementary file1 (PDF 318 KB)

## Data Availability

The JMDC claims data were used under contract with JMDC Inc. and cannot be freely distributed by the authors. However, data can be obtained directly from JMDC Inc. (https://www.jmdc.co.jp/en/jmdc-claims-database/). Only publicly available HBCR and PBCR data were used in this study.
