# Supplementary material for: Regional disparities in breast cancer healthcare in Japan: REAL-BC study
Source: Breast Cancer. 2025 Aug 2;32(5):1102–14. doi: 10.1007/s12282-025-01739-x (PMC12394363; doi:10.1007/s12282-025-01739-x)

ELECTRONIC SUPPLEMENTARY MATERIALS: ONLINE RESOURCE 1

Breast Cancer

Regional disparities in breast cancer healthcare in Japan: REAL-BC study

Hiroshi Kitagawa, Kazuki Fukuzawa, Masaki Tanaka

Corresponding author

Hiroshi Kitagawa

Medical Department, AstraZeneca K.K., Osaka, Japan

E-mail: hiroshi.kitagawa@astrazeneca.com

**Table of contents**	**Page**
**ESM Table 1.** Objectives, endpoints, and definitions	2
**ESM Table 2.** Functions, staffing, and medical service indicators	4
**ESM Table 3.** Sources of publicly available data	6
**ESM Table 4.** International Classification of Disease, 10th revision, diagnostic codes	8
**ESM Table 5.** Breast cancer treatments used to extract patients from the JMDC claims database	9
**ESM Table 6.** Number of DCCHs and proportion of DCCHs visited by breast cancer patients for initial treatment in 2018, 2019, and 2020 (HBCR database)	12
**ESM Table 7.** Number of breast cancer patients per DCCH, by prefecture (JMDC claims database)	12
**ESM Table 8.** Ratios of breast cancer patients who visited DCCHs for initial treatment by prefecture in 2020	13
**ESM Table 9.** Proportions of breast cancer patients visiting DCCHs for initial treatment, per calendar year (PBCR and HBCR databases)	15
**ESM Figure 1.** Bar chart showing the proportions of patients registered in the HBCR out of all breast cancer patients registered in the PBCR, and the proportions of patients registered in the HBCR who visited a DCCH	16

ESM Table 1. Objectives, endpoints, and definitions

Outcome	Objective	Endpoint	Definition/analysis
Primary	To determine the prefectural differences in ratios of breast cancer patients visiting DCCHs for their initial treatment in 2020	Ratio of breast cancer patients registered in DCCHs and each prefecture in 2020 by HBCR and PBCR	Ratio = (number of breast cancer patients visiting DCCHs for their initial treatment)/(number of breast cancer patients registered in PBCR) × 100
Secondary	To determine the prefectural reality in terms of the functions, staffing, and medical services in DCCHs for breast cancer patients	Proportion of DCCHs with a specific function	Proportion = (number of DCCHs with a specific function)/(number of DCCHs) × 100
		Number of specific staff	Number of breast cancer patients per specific staff in DCCHs
		Proportion of DCCHs with breast cancer patients who received a specific medical service	Proportion = (Number of DCCHs with breast cancer patients who received a specific medical service)/(Number of DCCHs) × 100
Exploratory	To determine the regional (secondary healthcare service area unit) differences in proportions of breast cancer patients visiting DCCHs for their initial treatment in 2020	Proportion of breast cancer patients registered in DCCHs for secondary healthcare service area unit in 2020	Proportion = (number of breast cancer patients visiting DCCHs for their initial treatment)/(number of breast cancer patients registered in HBCR) × 100

	To determine the trends of prefectural differences in ratios of breast cancer patients visiting DCCHs for their initial treatment each calendar year from 2018 to 2020	Ratio of breast cancer patients registered in DCCHs from 2018 to 2020 by HBCR and PBCR	Same as primary objective

DCCH designated cancer care hospital, *HBCR* hospital-based cancer registry; *PBCR* population-based cancer registry

ESM Table 2. Function, staffing, and medical service indicators

Category	Indicator	Period
Functions [a]	Proportion of JBCS-certified hospitals	2020
	Proportion of JBCS-affiliated hospitals	2020
	Proportion of designated Cancer Genome Medicine core hospitals	2020
	Proportion of JOPBS implantation-certified hospitals	2020
Staffing [a]	Number of breast cancer patients per JBCS-certified physician	2021
	Number of breast cancer patients per JOPBS-certified responsible physician and performing physician	2024
	Number of breast cancer patients per JSMO-certified medical oncologist	2021
	Number of breast cancer patients per JSP-certified pathologist	2021
	Number of breast cancer patients per JASTRO/JRS-certified radiologist	2021
	Number of breast cancer patients per JSPHCS-certified cancer specialist pharmacist	2021
	Number of breast cancer patients per JNA-certified breast cancer nurse	2021
Services [b]	Proportion of DCCHs with breast cancer patients with medical claims for “Cancer patient guidance and management fee (I)” *Explanation:* The physician discusses the treatment strategy with a nurse and provides a written document explaining the treatment	2019 & 2020
	Proportion of DCCHs with breast cancer patients with medical claims for “cancer patient guidance and management fee (Ro)” *Explanation*: A physician, nurse, or certified clinical psychologist conducts interviews to reduce the patient’s psychological anxiety	2019 & 2020
	Proportion of DCCHs with breast cancer patients with medical claims for “cancer patient guidance and management fee (Ha)” Explanation: A physician or pharmacist explains, in writing, the necessity for administering anticancer agents	2019 & 2020

Proportion of DCCHs with breast cancer patients with medical claims for "cancer patient guidance and management fee (Ni)" *Explanation:* A physician explains the necessity, in writing, for genetic testing	2020
Proportion of DCCHs with breast cancer patients with medical claims for "cancer genome profiling test fee"	2020
Proportion of DCCHs with breast cancer patients with medical claims for "cancer *BRCA1/2* genetic test fee"	2020
Proportion of DCCHs with breast cancer patients with medical claims for "genetic counseling fee"	2020
Proportion of DCCHs with breast cancer patients with medical claims for "patient support system enhancement fee"	2019 & 2020
Proportion of DCCHs with breast cancer patients with medical claims for "cancer patient rehabilitation fee"	2019 & 2020
Proportion of DCCHs with breast cancer patients with medical claims for "lymphedema combination therapy fee"	2019 & 2020
Proportion of DCCHs with breast cancer patients with medical claims for "cancer treatment coordination planning fee 1"	2019 & 2020

[a] See **ESM Table 3** for sources of data; DCCHs were identified from a list dated April 1, 2021

[b] Extracted from the JMDC Claims Database using relevant medical service fees; DCCHs were identified from a list dated April 1, 2023

DCCH designated cancer care hospital, *JASTRO/JRS* Japanese Society for Radiation Oncology/Japan Radiological Society, *JBCS* Japanese Breast Cancer Society, *JNA* Japanese Nursing Association, *JOPBS* Japan Oncoplastic Breast Surgery Society, *JSMO* Japanese Society of Medical Oncology, *JSP* Japanese Society of Pathology, *JSPHCS* Japanese Society of Pharmaceutical Health Care and Sciences

ESM Table 3. Sources of publicly available data

Item	Data source
JBCS-certified/affiliated hospitals	https://web.archive.org/web/20200503065540/http:/jbcs.gr.jp/member/aboutus/shisetsu/ (Access date: 2 May 2024)
JBCS-certified physicians	2021 DCCH status reports or recommendation for new designation or renewal of designation (Access date: 3 June 2024)
JOPBS implantation-certified hospitals	https://web.archive.org/web/20191029065104/http://jopbs.umin.jp/common/shisetsu/jisshi_shisetsu_nintei_imp.pdf (Access date: 7 May 2024)
JOPBS-certified responsible physicians and performing physicians	http://jopbs.umin.jp/medical/doctor/sekinin.html http://jopbs.umin.jp/medical/doctor/jisshi.html (Access date: 7 May 2024)
JSMO-certified medical oncologists	2021 DCCH status reports or recommendation for new designation or renewal of designation (Access date: 3 June 2024)
JSP-certified pathologists	2021 DCCH status reports or recommendation for new designation or renewal of designation (Access date: 3 June 2024)
JASTRO/JRS-certified radiologists	2021 DCCH status reports or recommendation for new designation or renewal of designation (Access date: 3 June 2024)
JSPHCS-certified cancer specialist pharmacists	2021 DCCH status reports or recommendation for new designation or renewal of designation (Access date: 3 June 2024)
JNA-certified breast cancer nurses	2021 DCCH status reports or recommendation for new designation or renewal of designation (Access date: 3 June 2024)

DCCH designated cancer care hospital, *JASTRO/JRS* Japanese Society for Radiation Oncology/Japan Radiological Society, *JBCS* Japanese Breast Cancer Society, *JNA* Japanese

Nursing Association, *JOPBS* Japan Oncoplastic Breast Surgery Society, *JSMO* Japanese Society of Medical Oncology, *JSP* Japanese Society of Pathology, *JSPHCS* Japanese Society of Pharmaceutical Health Care and Sciences

ESM Table 4. International Classification of Disease, 10th revision, diagnostic codes

Code	Diagnosis
C50.0	Nipple and areola
C50.1	Central portion of breast
C50.2	Upper-inner quadrant of breast
C50.3	Lower-inner quadrant of breast
C50.4	Upper-outer quadrant of breast
C50.5	Lower-outer quadrant of breast
C50.6	Axillary tail of breast
C50.8	Overlapping lesion of breast
C50.9	Breast, unspecified
D05.0	Lobular carcinoma in situ
D05.1	Ductal carcinoma in situ
D05.7	Carcinoma in situ, other
D05.9	Carcinoma in situ, unspecified

ESM Table 5. Breast cancer treatments used to extract patients from the JMDC claims database

Surgery

Category	Medical procedure
Partial mastectomy	Partial mastectomy
Mastectomy	Mastectomy<br>Mastectomy (patients with hereditary breast or ovarian cancer)
Destruction of breast lesion	Destruction of breast lesion
Mammary gland malignancy surgery	Simple mastectomy (total mastectomy)<br>Simple bilateral mastectomy with lymph node radical excision<br>Lumpectomy with axillary dissection<br>Extended radical bilateral mastectomy<br>Partial mastectomy (with axillary lymph node radical excision)<br>Partial mastectomy (without axillary lymph node radical excision)<br>Mastectomy (without axillary dissection)<br>Nipple-sparing mastectomy (without axillary dissection)<br>Nipple-sparing mastectomy (with axillary dissection)<br>Mammary gland malignancy surgery and bilateral lymph node dissection

Radiotherapy

Medical act
Radiation therapy management fee (one-beam irradiation)
Radiation therapy management fee (opposed two-port irradiation)
Radiation therapy management fee (external irradiation)
Radiation therapy management fee (non-opposed two-port irradiation)
Radiation therapy management fee (three-port irradiation)
Radiation therapy management fee (intracavitary irradiation)
Radiation therapy management fee (four-port irradiation)
Radiation therapy management fee (whole-body irradiation)
Radiation therapy management fee (motion irradiation)

Radiation therapy management fee (intratissue irradiation)
Radiation therapy management fee (intensity-modulated radiation therapy)
Radiation therapy management radiation therapy dedicated fee
Outpatient radiation therapy fee
Remote radiation therapy planning fee
External beam radiation therapy (high-energy radiation therapy) (single-port irradiation) [a]
External beam radiation therapy (high-energy radiation therapy) (opposed two-port irradiation) [a]
External beam radiation therapy (high-energy radiation therapy) (non-opposed two-port irradiation) [a]
External beam radiation therapy (high-energy radiation therapy) (three-port irradiation) [a]
External beam radiation therapy (high-energy radiation therapy) (irradiation with four or more ports) [a]
External beam radiation therapy (high-energy radiation therapy) (motion irradiation) [a]
External beam radiation therapy (high-energy radiation therapy) (whole-body irradiation) [a]
Facility standard non-compliance deduction (radiation) (70 out of 100)
Image-guided radiation therapy fee (surface position information)
Image-guided radiation therapy fee (bone structure position information)
Image-guided radiation therapy fee (tumor position information)
Radiation therapy with gamma knife
Radiation therapy with linear accelerator (stereotactic radiation therapy)
Radiation therapy with linear accelerator (non-first session)
Radiation therapy with linear accelerator (stereotactic radiation therapy for the trunk)
Stereotactic radiation therapy respiratory motion management fee (motion tracking method)
Stereotactic radiation therapy respiratory motion management fee (other methods)

[a] First or second session

Pharmacotherapies

**Generic name**		
Abemaciclib	Everolimus	Olaparib
Aclarubicin hydrochloride	Exemestane	Paclitaxel
Anastrozole	Fluorouracil	Palbociclib
Atezolizumab	Fulvestrant	Pembrolizumab
Bevacizumab	Gemcitabine hydrochloride	Pertuzumab
Capecitabine	Goserelin acetate	Pirarubicin hydrochloride
Carboplatin	Irinotecan hydrochloride	Tamoxifen citrate
Cyclophosphamide	Lapatinib ditosylate	Tegafur and uracil
Cytarabine	Letrozole	Tegafur, gimeracil and oteracil potassium (S-1)
Docetaxel	Leuprolide acetate	Toremifene citrate
Doxifluridine	Medroxyprogesterone acetate	Trastuzumab
Doxorubicin hydrochloride	Mepitiostane	Trastuzumab deruxtecan
Epirubicin hydrochloride	Methotrexate	Trastuzumab emtansine
Eribulin mesylate	Mitomycin	Vinorelbine tartrate
Ethinyl estradiol	Mitoxantrone hydrochloride	

ESM Table 6. Number of DCCHs and proportion of DCCHs visited by breast cancer patients for initial treatment in 2018, 2019, and 2020 (HBCR database)

	**DCCHs, *n***			**DCCHs visited by ≥1 breast cancer patients for their initial treatment, *n* (%)**		
	**2018**	**2019**	**2020**	**2018**	**2019**	**2020**
Total	433	446	450	430 (99.3)	444 (99.6)	448 (99.6)

DCCH designated cancer care hospital, *HBCR* hospital-based cancer registry

ESM Table 7. Number of breast cancer patients per DCCH, by prefecture (JMDC claims database)

	**Number of DCCHs** [a]	**Number of breast cancer patients per DCCH**				
		**Mean**	**SD**	**Minimum**	**Median**	**Maximum**
Total	389	50.3	67.66	1	29	599

[a] DCCHs where ≥1 breast cancer patient received any breast cancer treatment

DCCH designated cancer care hospital, *SD* standard deviation

ESM Table 8. Ratios of breast cancer patients who visited DCCHs for initial treatment by prefecture in 2020

Prefecture	Number of patients with breast cancer [a]	Number of patients with breast cancer patients who received initial treatment (%) [b]	Number of patients with breast cancer who visited a DCCH for their initial treatment (%) [b]
Total	103744	79062 (76.2)	59891 (57.7)
Hokkaido	4692	3207 (68.4)	2224 (47.4)
Aomori	1090	876 (80.4)	524 (48.1)
Iwate	1006	725 (72.1)	699 (69.5)
Miyagi	1954	1208 (61.8)	1155 (59.1)
Akita	755	665 (88.1)	470 (62.3)
Yamagata	809	665 (82.2)	553 (68.4)
Fukushima	1419	934 (65.8)	563 (39.7)
Ibaraki	2369	1802 (76.1)	1370 (57.8)
Tochigi	1560	1391 (89.2)	1063 (68.1)
Gunma	1521	1508 (99.1)	1366 (89.8)
Saitama	5986	3570 (59.6)	2568 (42.9)
Chiba	5080	3872 (76.2)	2910 (57.3)
Tokyo	11698	11086 (94.8)	8522 (72.9)
Kanagawa	7670	5310 (69.2)	4032 (52.6)
Niigata	1847	1526 (82.6)	1285 (69.6)
Toyama	886	613 (69.2)	419 (47.3)
Ishikawa	962	858 (89.2)	654 (68.0)
Fukui	553	517 (93.5)	477 (86.3)
Yamanashi	678	445 (65.6)	445 (65.6)
Nagano	1586	1254 (79.1)	1168 (73.6)
Gifu	1509	1025 (67.9)	879 (58.3)
Shizuoka	3012	2571 (85.4)	2034 (67.5)
Aichi	5634	4393 (78.0)	3482 (61.8)

Mie	1439	1185 (82.3)	749 (52.1)
Shiga	1008	681 (67.6)	426 (42.3)
Kyoto	2128	1461 (68.7)	1127 (53.0)
Osaka	7277	6335 (87.1)	3292 (45.2)
Hyogo	4617	3169 (68.6)	2346 (50.8)
Nara	1109	850 (76.6)	677 (61.0)
Wakayama	751	452 (60.2)	414 (55.1)
Tottori	483	456 (94.4)	242 (50.1)
Shimane	485	404 (83.3)	355 (73.2)
Okayama	1613	1308 (81.1)	991 (61.4)
Hiroshima	2242	2061 (91.9)	1993 (88.9)
Yamaguchi	1085	579 (53.4)	517 (47.6)
Tokushima	592	494 (83.4)	440 (74.3)
Kagawa	764	404 (52.9)	404 (52.9)
Ehime	1200	1086 (90.5)	973 (81.1)
Kochi	626	438 (70.0)	293 (46.8)
Fukuoka	4379	2921 (66.7)	2710 (61.9)
Saga	634	254 (40.1)	252 (39.7)
Nagasaki	1167	819 (70.2)	657 (56.3)
Kumamoto	1526	1186 (77.7)	476 (31.2)
Oita	900	404 (44.9)	351 (39.0)
Miyazaki	927	295 (31.8)	205 (22.1)
Kagoshima	1294	1024 (79.1)	950 (73.4)
Okinawa	1222	775 (63.4)	189 (15.5)

[a] Patients with breast cancer who were registered with the PBCR

[b] The number of patients registered with the HBCR was used as the numerator, and the number of patients registered with the PBCR was used as the denominator

DCCH designated cancer care hospital, *HBCR* hospital-based cancer registry, *PBCR*, population-based cancer registry

ESM Table 9. Proportions of breast cancer patients visiting DCCHs for initial treatment, per calendar year (PBCR and HBCR databases)

**Year**	**Ratio of breast cancer patients visiting DCCHs for their initial treatment (%)** [a]					
	n	**Mean**	**SD**	**Minimum**	**Median**	**Maximum**
2018	47	54.48	14.965	16.8	53.38	82.3
2019	47	56.86	14.660	17.0	57.17	85.7
2020	47	57.63	15.860	15.5	57.83	89.8

[a] Breast cancer patients who were registered with the HBCR was used as the numerator. Breast cancer patients who were registered with the PBCR was used as the denominator

DCCH designated cancer care hospital, *HBCR* hospital-based cancer registry, *PBCR* population-based cancer registry, *SD* standard deviation

ESM Figure 1. Bar chart showing the proportions of patients registered in the HBCR out of all breast cancer patients registered in the PBCR, and the proportions of patients registered in the HBCR who visited a DCCH. *DCCH* designated cancer care hospital, *HBCR* hospital-based cancer registry, *PBCR* population-based cancer registry

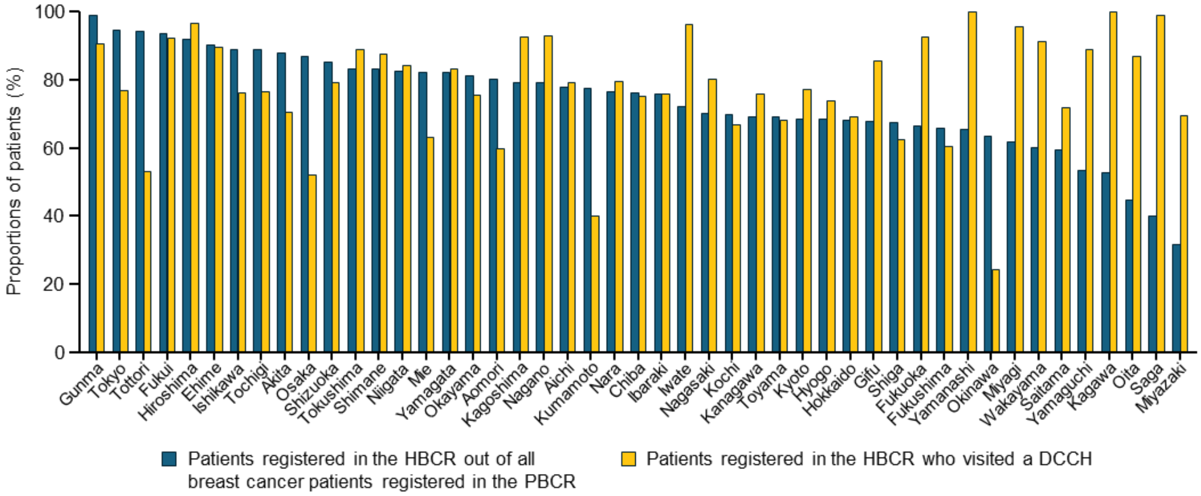

Supplement: Supplementary file 1 — Supplementary file1 (PDF 318 KB) [file 12282_2025_1739_MOESM1_ESM.pdf]
